# Microstructure, Precipitates Behavior, and Mechanical Properties of Age-Hardened Al-Mg-Si Alloy Sheet Fabricated by Twin-Roll Casting

**DOI:** 10.3390/ma15165638

**Published:** 2022-08-16

**Authors:** Guanjun Gao, Xiwu Li, Baiqing Xiong, Zhihui Li, Yongan Zhang, Yanan Li, Lizhen Yan

**Affiliations:** 1 State Key Laboratory of Non-Ferrous Metals and Processes, GRINM Group Co., Ltd., Beijing 100088, China; 2 GRIMAT Engineering Institute Co., Ltd., Beijing 101407, China; 3 General Research Institute for Nonferrous Metals, Beijing 100088, China

**Keywords:** Al-Mg-Si alloy, second particles, texture, precipitates behavior, twin-roll casting

## Abstract

Twin-roll casting (TRC), as a near-net-shape technology, is employed to fabricate age-hardened Al-Mg-Si alloy. Compared with conventional direct chill (DC) casting, the TRC method is much more economical and efficient. In this work, the microstructure, precipitates behavior, and mechanical properties of age-hardened Al-Mg-Si alloy sheet fabricated by TRC were investigated by hardness measurements and tensile tests, metallographic microscopy, field emission gun scanning electron microscope, electron backscatter diffraction, transmission electron microscopy, and differential scanning calorimetry analyses. It was found that the size of recrystallized grains for DC casting alloy with finely dispersed particles was larger than that of TRC alloy with coarse particles. Typical Cube_ND_ texture accompanied by P texture formed after solution treatment made the value of *r* reach ~0.7 in the TRC alloy due to the PSN effect caused by the segregation of particles. More GP zones resulted in the strength of TRC alloy being higher than that of DC casting alloy after T8X treatment. With the time of paint-bake hardening extended to 8 h, few segregation particles remained in the TRC alloy. This decreased the concentration of supersaturated atoms. The hardness of the TRC alloy with the lower density of the β″ strengthening phase was lower compared to the DC casting alloy.

## 1. Introduction

**Figure 1:**
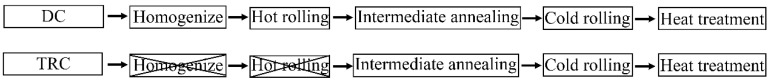
Schematic diagram of DC and TRC process of Al-Mg-Si alloy sheet.

Due to their high strength-to-weight ratio, good corrosion resistance, and excellent ductility, Al-Mg-Si alloy sheets continue to draw great attention from the automotive industry [1,2]. Twin-roll casting (TRC) as a near-net-shape technology is much more economical than the conventional method for Al-Mg-Si alloy preparation. Compared with conventional direct chill (DC) casting, the TRC method decreases the technological processing time, reduces energy consumption, requires less equipment, and has a lower running cost. The huge energy and processing time consumption of homogenization treatment and hot-rolling may be removed by TRC technology, as shown in Figure 1. Obviously, TRC technology can be used to optimize the industrial production process of Al-Mg-Si alloy sheets with acceptable microstructure and mechanical properties [3,4,5].

There are inevitable defects, such as centerline macro segregation or dendritic segregation in the Al-Mg-Si alloy sheets produced by the TRC method. These casting defects are not effectively eliminated by the subsequent hot/cold-rolling and heat-treatment processes and further affect the performance of the final alloy sheets. However, the positive effects of the microstructure, precipitates behavior, and mechanical properties are visible through controlling the TRC process and subsequent heat-treatment processes in the present study. Lu et al. [6] revealed that the homogenization process significantly affected second particles by promoting dissolution of Mg_2_Si phases, the transformation of the Fe-rich intermetallic phase (β-AlFeSi is transformed into discrete α-Al(FeMn)Si particles with a rounded shape). Coarse intermetallic phases and fine dispersoids were achieved. Favorable recrystallization texture intensity and components were gained and improved the deep drawability of the Al-Mg-Si alloy sheets. A few studies [5] revealed that the introduction of external fields in TRC has great potential for the improvement of segregation. When a static magnetic field and pulsed current field were applied, the electromagnetic braking effect and shock wave effect resulted in the uniformity of the microstructure, the composition distribution was improved, and the mixing capacity and solid solubility of the alloy elements were increased. This reduced the difference in the hardness in the thickness direction and the mechanical properties in the width direction. Li et al. [7] revealed that the secondary dendrite arm spacing (SDAS) of the alloy was significantly reduced, and the solidification structure of the alloy was dramatically refined by electromagnetic twin-roll casting (ETRC) technology. The volume fraction of non-equilibrium eutectic phases, intermetallic, which are continuously distributed in the interdendritic region and grain boundaries, remarkably decreased. This resulted in the homogenization time of the alloy fabricated by ETRC being greatly shortened compared with traditional methods. The alloying elements also obviously affected the microstructure of the TRC Al-Mg-Si alloy and, furthermore, affected the mechanical properties of the alloy [8,9,10,11]. The low Mg/Si ratio promoted precipitation during aging, which resulted in an increase in yield strength compared to the alloy with Mg/Si = 2.9. However, intergranular fracture occurred in the alloy with Mg/Si = 0.5, lowering uniform elongation [8]. The strength and hardness of the TRC 6061 alloy increased first and then decreased with the increase in Mn content in the alloy, and the elongation decreased gradually with the increase in Mn content [9]. The Fe-bearing phase plays a significant role in the mechanical properties of the TRC Al-Mg-Si alloy. The decomposition of the π-AlFeMgSi phase expanded the solid solubility and promoted aging precipitation, resulting in higher yield strength of the alloy during the peak-aging treatment [10]. Grain refinement was probably attributed to the Cr-addition-induced formation of Al_45_Cr_7_ phases acting as heterogeneous nucleation sites for the alloy. Tensile properties of solution-treated (T4) and peak-aged (T6) alloys were synchronously optimized [11].

Compared to conventional DC casting, the TRC method has special advantages. To the best of our knowledge, little is known concerning the TRC Al-Mg-Si alloy sheets. In this study, the corresponding microstructure, precipitates behavior, and mechanical properties of an age-hardened Al-Mg-Si alloy sheet fabricated by conventional DC casting, TRC, with and without homogenization heat treatment, are revealed in detail. We hope that the obtained findings are used to enhance indispensable information for optimizing the production of high-quality and low-cost automotive Al-Mg-Si alloy sheets using TRC technology.

## 2. Materials and Methods

**Figure 2:**
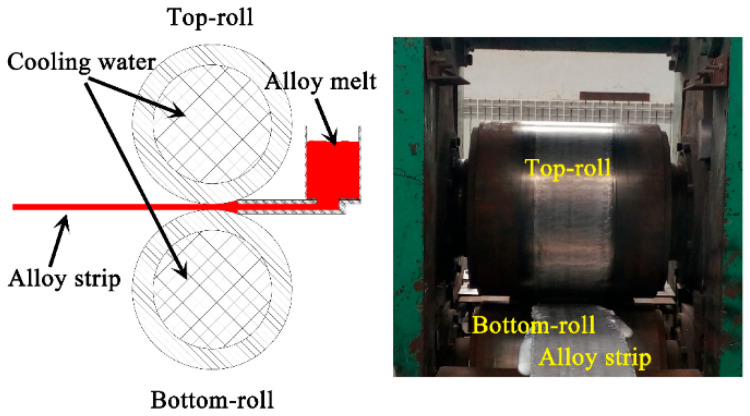
Schematic illustration of TRC technique and physical map of TRC experimental device.

As shown in Figure 2, a horizontal twin-roll caster with a maximum rolling force of 1000 KN was used in this study. The diameter of the top roll and bottom roll with cooling water is 500 mm. The roller gap was set to 6 mm. Graphite was used to coat the roller surface to avoid sticking of the strip. The Al-Mg-Si alloy with a chemical composition of Al-1.17%, Si-0.52%, Mg-0.18%, and Fe-0.10% Cr (wt.%) was fabricated by experimental TRC device. The started temperature of cast rolling and the initial rolling speed was 690 °C and 1.1 m/min, respectively.

For the Al-Mg-Si alloy sheet fabricated by TRC method, a group of TRC sheet intermediates annealed at 350 °C for 2 h was cold-rolled from 6 mm to 1 mm (A2 alloy). The other group was directly cold-rolled after homogenization treatment at 540 °C for 8 h. The final cold-rolling thickness of the sheet was 1 mm (A3 alloy). A commercial Al-Mg-Si alloy sheet cold-rolled to 1 mm in thickness (A1 alloy) was used for comparison in this study. The above three groups of alloy sheets were subjected to solution treatment at 560 °C for 10 min, followed by water quenching to room temperature (RT). The quenched alloy sheets were immediately pre-aged at 100 °C for 3 h (T4P state). Two weeks of RT storage and 2% tensile deformation for the alloy sheets (simulating the transportation and stamping process of automotive sheet) was introduced before paint-bake hardening treatment at 185 °C.

## 3. Results

### 3.1. Second Particles

**Figure 3:**
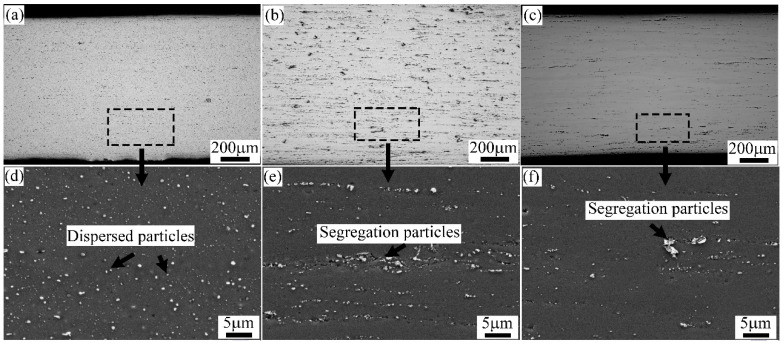
OM and SEM images of the second particles’ distribution in the cold-rolled alloy sheets: (**a**,**d**) A1 alloy, (**b**,**e**) A2 alloy, and (**c**,**f**) A3 alloy.

Figure 3 shows OM and SEM images of the second particles’ distribution of the cold-rolled alloy sheets. As can be seen in Figure 3, the finely dispersed particles are uniformly distributed on the Al-Mg-Si alloy prepared by conventional DC casting (A1 alloy), while the segregated and dispersed particles linearly distribute along the rolling direction in the TRC alloy (A2 alloy). Some small-sized second particles were redissolved in the alloy matrix after short-time homogenization treatment (A3 alloy). Obviously, the number of second particles in the A3 alloy was lower than that in the first two groups of alloys.

**Figure 4:**
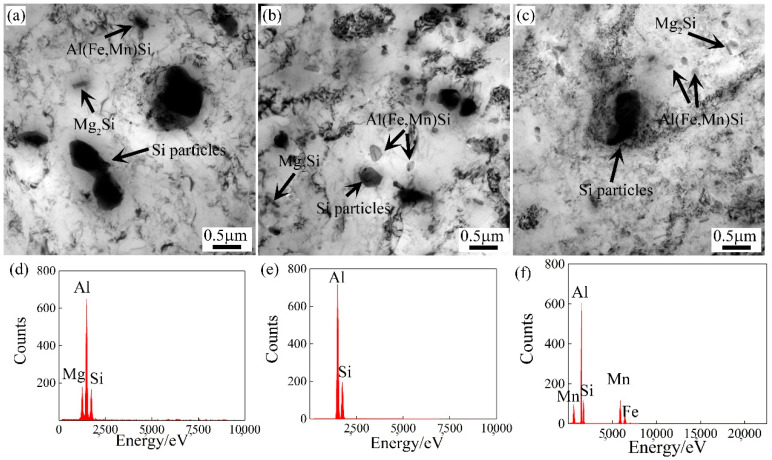
TEM images and EDS analysis of the second particles’ distribution in the cold-rolled alloy sheets: (**a**,**d**) A1 alloy, (**b**,**e**) A2 alloy, and (**c**,**f**) A3 alloy.

TEM images and EDS analysis of the second particles’ distribution in the cold-rolled alloy sheets are shown in Figure 4. The results demonstrated that the A1, A2, and A3 alloys exhibited three types of second phases. Based on EDS analysis, the black blocky phase was Si particles. The gray particles were Mg_2_Si and α-Al(Fe,Mn)Si particles, respectively. The rod-shaped Mg_2_Si and round-shaped α-Al(Fe,Mn)Si particles are dominant in the A1 alloy. The segregation phase in A2 and A3 alloys were primarily mixed Mg_2_Si and Si particles [12]. Compared with the A1 alloy, the number of primary Si particles was significantly reduced.

### 3.2. Microstructure and Texture

**Figure 5:**
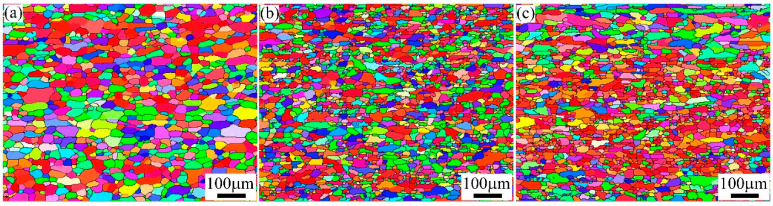
Recrystallized structure of A1, A2, and A3 alloys after solution treatment: (**a**) A1 alloy, (**b**) A2 alloy, and (**c**) A3 alloy.

Figure 5 shows the recrystallized structure of A1, A2, and A3 alloys after solution treatment. It can be noted that complete recrystallization occurred in the alloys after solution treatment, and nearly equiaxed grains were formed. For the A1 alloy, recrystallized grains with an average size of 25.2 μm were formed during solution treatment. The average grain sizes of A2 and A3 alloys were calculated as 16.9 μm and 18.1 μm, respectively. It can be observed that the grain size distribution of A1 and A2 alloys was relatively uniform. In contrast to the A1 and A2 alloys, the A3 alloy had a recrystallized structure with a large difference in grain size.

**Figure 6:**
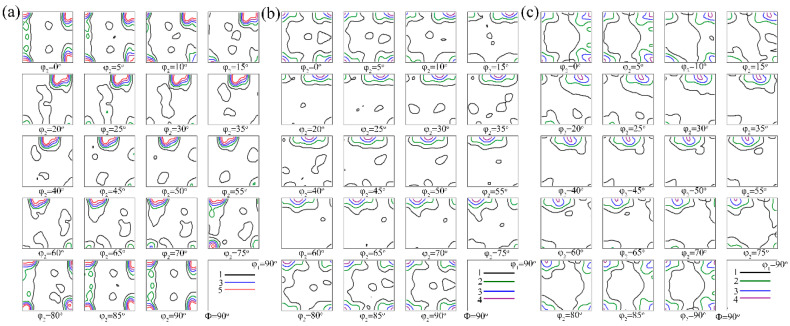
The ODF maps showing recrystallization texture of A1, A2, and A3 alloys after solution treatment: (**a**) A1 alloy, (**b**) A2 alloy, and (**c**) A3 alloy.

Figure 6 shows the orientation distribution function (ODF) maps of A1, A2, and A3 alloys after solution treatment. It was used to calculate the intensities and volume fractions of the recrystallization texture components. The values listed in Table 1 clearly indicated that the Cube texture component was the main recrystallization texture in three groups of alloys. The intensity and volume fraction of the Cube component in the A1 alloy was higher than that in A2 and A3 alloys. The A2 and A3 alloys contained Cube_ND_ in addition to the Cube component. The volume fraction of the Cube_ND_ texture component was 12.9% and 12.7%, respectively. In addition, other texture components, such as P {011} <122> and H {001} <110> components, were formed in A2 and A3 alloys during solution treatment.

### 3.3. Precipitation Behavior

**Figure 7:**
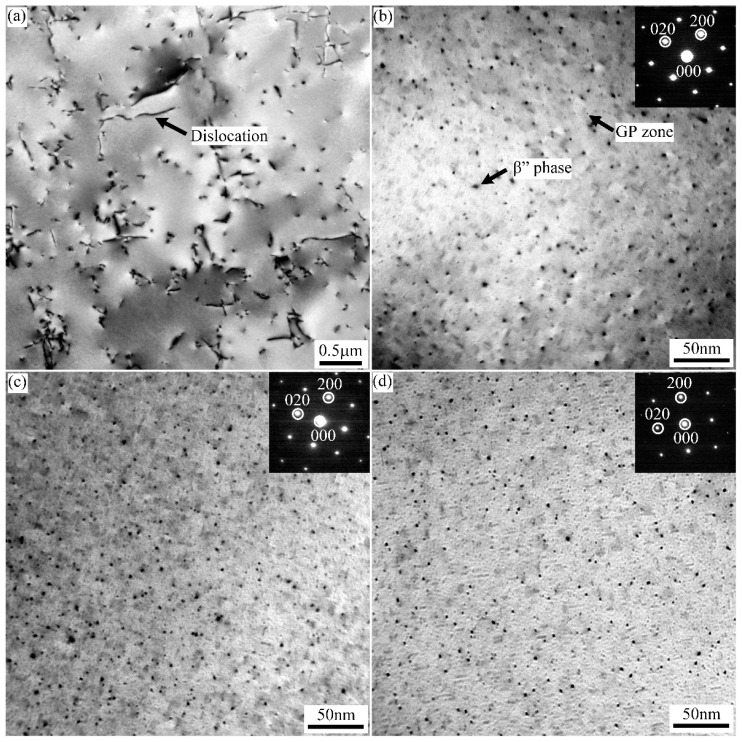
TEM bright field images of A1, A2, and A3 alloys after T8X treatment: (**a**) dislocations introduced by 2% tensile deformation; (**b**–**d**) strengthened precipitates of A1, A2, and A3 alloys.

The TEM bright field images of A1, A2, and A3 alloys treated with 2% tensile deformation before paint-bake hardening at 185 °C for 20 min (T8X treatment) are shown in Figure 7. A large number of dislocations introduced by 2% tensile deformation were distributed in the alloy matrix. The TEM images of strengthened precipitates were taken with the electron beam parallel along the [001]_Al_ zone axis. In the three groups of alloys, a number of fine dot-like and needle-like precipitates were observed. The dot-like precipitates appeared needle-like when viewed end-on in another direction. Specifically, the strengthened precipitates density in A1 alloy was lower than that of A2 and A3 alloys.

**Figure 8:**
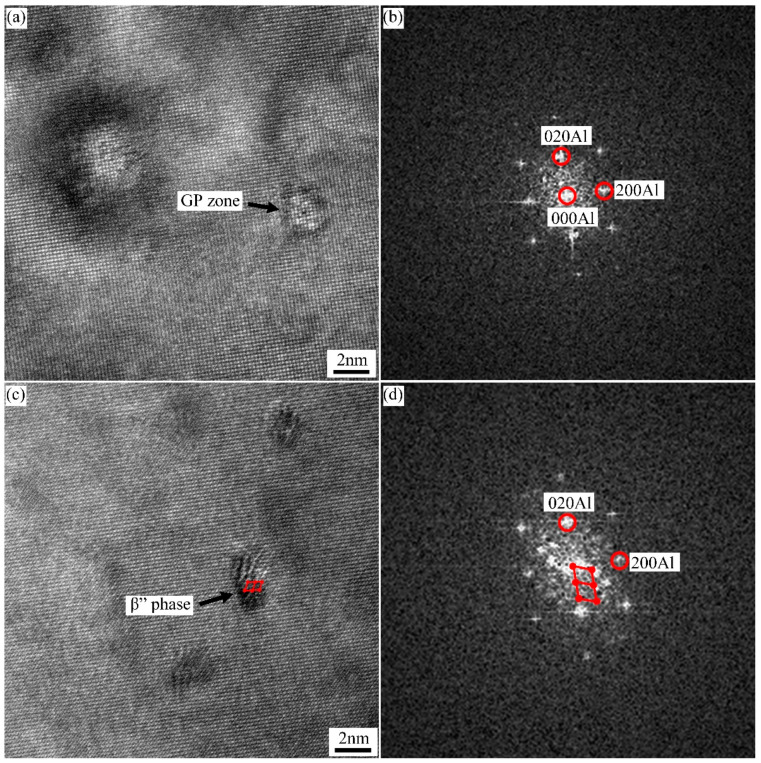
HRTEM images and corresponding FFT patterns of the precipitates marked with the arrows in Figure 7: (**a**,**b**) GP zone and (**c**,**d**) β″ precipitates.

The fine dot-like strengthened precipitates were further characterized by HRTEM images and corresponding FFT patterns, as shown in Figure 8. Two kinds of precipitates were formed in the three groups of alloys during T8X treatment. Some larger dot-like precipitates (~3–4 nm) were clearly observed in Figure 8c. These dot-like precipitates viewed end-on by needles were identified as β″ phase [13,14]. The β″ phase, with a semi-coherent orientation relationship with the Al matrix, caused severe lattice distortion. In contrast, the small precipitates with a size of ~2 nm were fully coherent with the Al matrix and did not cause atomic misalignment. According to the corresponding FFT patterns, neither extra reflection nor diffuse scattering was observed. These smaller precipitates were identified as the GP zone [15], which can serve as nucleation for β″ phase growth during paint-bake hardening treatment.

**Figure 9:**
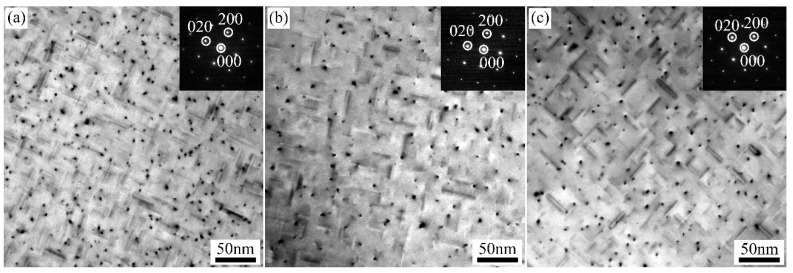
TEM bright field images of A1, A2, and A3 alloys after paint-bake hardening at 185 °C for 8 h: (**a**) A1 alloy, (**b**) A2 alloy, and (**c**) A3 alloy.

Figure 9 shows TEM bright field images of A1, A2, and A3 alloys after paint-bake hardening at 185 °C for 8 h. Compared with the alloys after T8X treatment, more needle-like β″ strengthened precipitates were observed in the Al matrix when extending the time of paint-bake hardening treatment. The number of β″ precipitates in the A1 alloy was significantly higher than that in A2 and A3 alloys. The distribution characteristics of β″ precipitates in three groups of alloys in Figure 9 were statistically analyzed. In order to ensure the accuracy of the statistical results, at least 1000 precipitates were counted. The statistical data of β″ precipitates listed in Table 2 show that the number density of 3.11 × 10^3^ N/μm^2^ of β″ precipitates in A1 alloy was the highest. At the same time, the values of the number density for A2 and A3 alloys were 2.06 × 10^3^ N/μm^2^ and 1.27 × 10^3^ N/μm^2^, respectively. The result of the volume fraction was consistent with the number density. Like the average length, the β″ precipitates of the A1 and A2 alloys were similar in size. The average length, 18.67 nm for β″ precipitates in the A3 alloy, was the shortest.

### 3.4. Mechanical Properties

**Figure 10:**
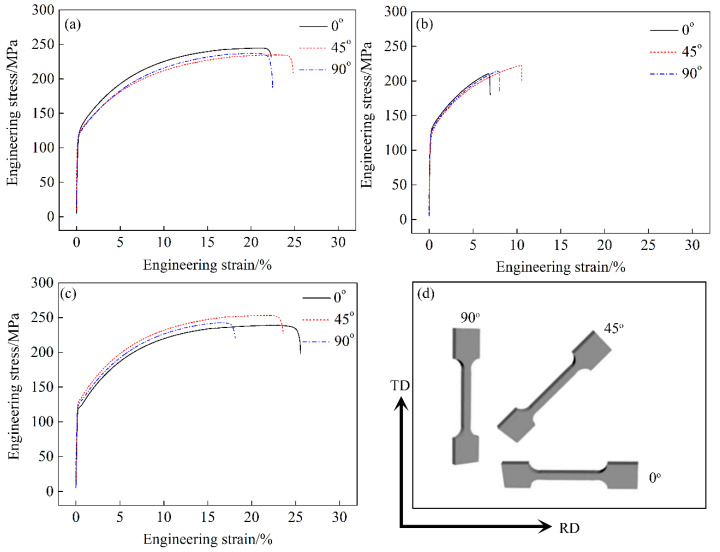
Engineering stress–strain curves of T4P-treated A1, A2 and A3 alloys in three directions: (**a**) A1 alloy, (**b**) A2 alloy, (**c**) A3 alloy, (**d**) Schematic diagram of the tensile direction.

Figure 10 shows the engineering stress–strain curves of T4P-treated alloys in three directions. The results revealed that the yield strength of the alloys with a T4P state reached ~135 MPa. It was worth noting that the engineering strain value of the A2 alloy in three directions was less than 15%, which was much lower than that in the other two groups of alloys.

**Figure 11:**
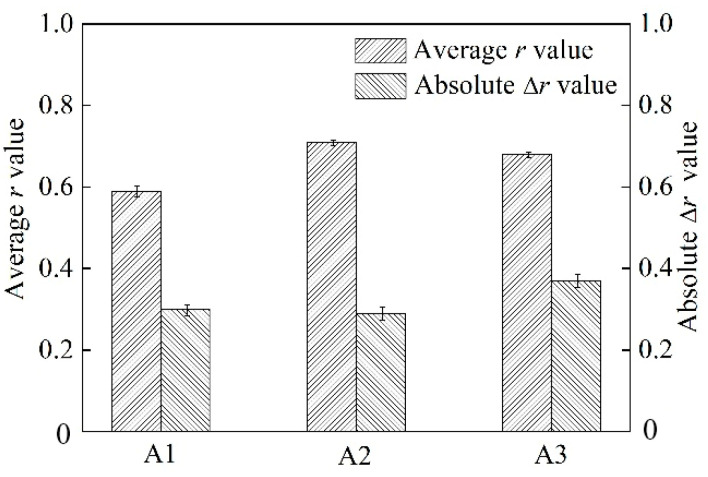
Average *r* and Δ*r* values of alloys with T4P state.

In general, the standard for automotive body sheets of aluminum alloy requires the *r* value (10% deformation) to be higher than 0.6. Meanwhile, the value of Δ*r* is preferably close to zero [16]. The average r and Δ*r* values were calculated using

(1) $$\bar{r}=\frac{r_{0^{o}}+2r_{45^{o}}+r_{90^{o}}}{4}\;\mathrm{and}\;\Delta r=\frac{r_{0^{o}}+r_{90^{o}}-2r_{45^{o}}}{2}$$

where $r_{0^{o}}$, $r_{45^{o}}$, and $r_{90^{o}}$ are the *r* values in three different directions. Figure 11 shows the average *r* and Δ*r* values of alloys with a T4P state. The average *r* value of A1, A2, and A3 alloy were 0.59, 0.71, and 0.68, respectively. The value of A1 was significantly lower than the A2 and A3 alloys. As for the Δ*r* value, the A1 and A2 alloys reached ~0.31. The Δ*r* value of the A3 alloy was ~0.37, which was a little higher than that of A1 and A2 alloys.

**Figure 12:**
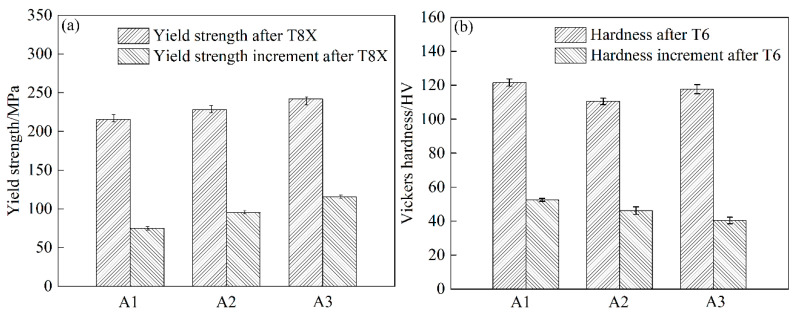
Yield strength and hardness increment of alloys after T8X and T6 treatment: (**a**) yield strength and (**b**) Vickers hardness.

Figure 12 shows the yield strength and hardness increment of alloys after T8X and T6 (paint-bake hardening at 185 °C for 8 h) treatment. As can be seen in Figure 12, the yield strength of the alloys with T8X treatment increased from A1 to A3. The values were about 215.2 MPa, 228.3 MPa, and 241.7 MPa, respectively. Moreover, the increment in yield strength was also increased, the same as yield strength. As for the hardness of alloys after T6 treatment, the A1 alloy with T6 treatment was the highest. The value of the hardness reached ~121.6 HV. The hardness of the A3 alloy was ~117.7 HV. The A2 alloy was the lowest, only about 110.5 HV. In addition, the hardness increment of the three groups of alloys decreased from the A1 to A3 alloy. The hardness increment of A1, A2, and A3 alloys after T6 treatment was about 52.5 HV, 46.2 HV, and 40.4 HV, respectively.

## 4. Discussion

Previous studies showed that the second particles in the alloy had a significant effect on the size and distribution of the recrystallized grain [17,18]. The movement of dislocations and the migration of sub-grain boundaries are significantly hindered by the high-density second particles in the A1 alloy. However, the nucleation rate of recrystallization decreased because of the small particle size, which resulted in coarse, recrystallized grains (average grain size ~25.2 μm, seen in Figure 5a). At the same time, the eutectic particles at the grain boundary of A2 alloy were broken to form coarse second particles after cold-rolling. These coarse particles can act as nucleate particles to increase the recrystallization nucleation rate and decrease the grain size (average grain size ~16.9 μm, seen in Figure 5b). As for the A3 alloy with a short homogenization treatment, the segregation in the matrix was obviously decreased. The number of coarse second particles and the density of nucleate particles decreased after cold-rolling. Therefore, the average grain size of the A3 alloy was slightly larger than that of the A2 alloy but still smaller than the A1 alloy (average grain size ~18.1 μm, seen in Figure 5c).

The size and distribution of the second particles in the cold-rolled sheet strongly affected the recrystallization texture during solution treatment [19,20,21]. Generally, the fine particles promoted nucleation on the cubic band, which resulted in the Cube texture being deflected to Goss orientation along the RD direction. Meanwhile, the large particles were beneficial to the PSN (particle-stimulated nucleation) effect and formed the random textures dominated by Cube_ND_ and P orientations [22]. The high-volume fraction of small particles resulted in stronger Zener drag. It also hindered the nucleation and growth of recrystallized grains. This led to the number of Cube textures formed during the recrystallization process. In addition, the strong Zener pinning force made the S orientation deflect to a weak angle during nucleation and the growth of grains, forming a small amount of R texture [23,24]. As for the A1 alloy, a large number of small particles existed in the matrix before solution treatment. Uniformly dispersed second particles greatly increased the Zener drag. This further resulted in the strong Cube and R texture formed after solution treatment (Figure 6a).

**Figure 13:**
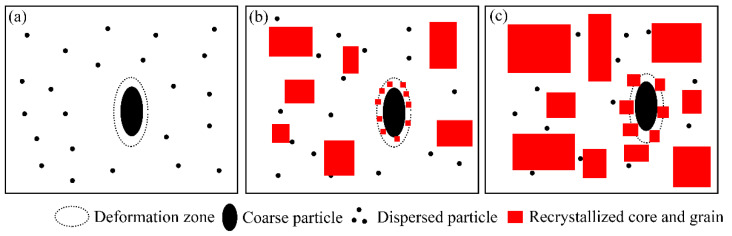
Schematic illustration of recrystallization during solution treatment: (**a**) before solution treatment, (**b**) initial stage of grain growth, and (**c**) post stage of grain growth.

Generally, a large number of coarse particles in the matrix caused PSN [23]. As shown in Figure 13, the grain cores with random orientation usually form in the deformation zone around coarse particles during solution treatment. In the initial stage of grain growth, these grain cores were more likely to grow into recrystallized grains towards Cube_ND_ and P orientations. Finally, typical Cube_ND_ and P texture components were formed. Therefore, the particles with a size in excess of *d*_crit_ will be able to initiate PSN

(2) $$d_{\mathrm{crit}}=\frac{4\gamma_{\mathrm{GB}}}{P_{D}-P_{Z}}$$

where *γ_GB_* is the specific grain boundary energy and *P*_D_ and *P*_Z_ are the driving pressure for recrystallization and the Zener drag due to second particles [21]. For the A2 alloy, the effect of PSN was significant due to a large number of segregation particles and coarse second phase particles that existed in the matrix. This resulted in the formation of typical Cube_ND_ and P textures (Figure 6b). The A3 alloy was homogenization-treated at 540 °C for 8 h. The number of coarse second particles (segregation particles) increased. Thus, the PSN effect of the A3 alloy was not significant. Only Cube_ND_ and H textures formed after solution treatment.

The previous study showed that the texture of an aluminum alloy is closely related to the *r* value [25,26]. The calculated $\bar{r}$ values of typical recrystallized texture components are listed in Table 3. The *r* value is 0.5 when only the Cube texture is formed in the alloy. At the same time, a high *r* value corresponds to Cube_ND_ and P texture components. The *r* value of the alloy with more than two typical textures is given by:

(3) $$\bar{r}=\sum V_{j}r_{j}$$

From Equation (3), a mean r value can be calculated by *j* oriented-grain corresponding to *r_j_* value and the volume fraction of *j* oriented-grains. A large number of small particles before solution treatment increased the Zener drag in the A1 alloy. The strong Cube and R texture formed further resulted in a low *r* value of the alloy with a T4P state. As for the A2 and A3 alloys, the effect of PSN was significant due to a large number of segregation particles and coarse second phase particles. The typical Cube_ND_ texture was accompanied by a P texture formed in the alloys. This made the *r* value of A2 and A3 alloys relatively higher (Figure 11).

**Figure 14:**
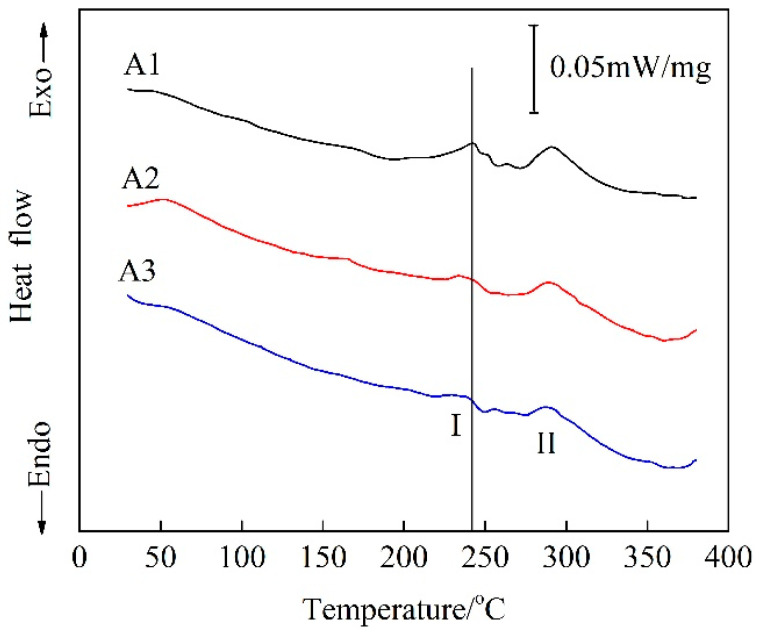
DSC curves of the alloys with PA treatment.

In order to analyze the precipitates behavior of the alloys, DSC curves at a heating rate of 10 °C/min from 35 °C to 380 °C for the alloys with PA treatment were carried out, as shown in Figure 14. Two exothermic peaks, peak I and peak II, corresponding to the formation of β″ and β′ phases, appeared on the curves [27,28]. With a close look at the DSC curves, one can observe that the temperature of the exothermic peak I for the A1 alloy was higher than that of A2 and A3 alloys. It can be inferred that the shorter paint-bake hardening time required for A2 and A3 alloys resulted in the formation of large numbers of β″ phases. In the typical paint-bake hardening process of aluminum alloy automotive sheets, the supersaturated solute atoms (raw materials) in the matrix were sufficient [29]. The strength of A2 and A3 alloys after T8X treatment was higher than that of the A1 alloy, causing more strengthening phases to be formed in the alloys with the same paint-bake hardening treatment condition [30,31]. In addition, the micro-segregation of the A3 alloy was decreased after a short homogenization treatment. More supersaturated solute Mg and Si atoms led to solution strengthening. This may be the reason why the strength of the A3 alloy was slightly higher than that of the A2 alloy [32,33].

When the time of paint-bake hardening for the three groups of alloys was extended to 8 h, most of the supersaturated solute atoms were consumed and transformed into strengthening precipitates. For the A1 alloy, the segregation in the alloy was eliminated after homogenization treatment at 540 °C for 20 h. A more soluble phase was dissolved in the matrix during solution treatment. As a result, the content of supersaturated solute atoms was the highest. The number density and volume fraction of the β″ phase were higher than those of the other two groups of alloys, resulting in the highest hardness of the alloy with a T6 state (Figure 9a and Figure 12b). The amount of segregation was maintained in the cold-rolled A2 alloy compared to the A1 alloy. The coarse segregation particles were not completely dissolved in the matrix after solution treatment. The concentration of supersaturated Mg and Si atoms decreased, which led to a significant decrease in the number of β″ phases (Figure 9b) [34,35]. As for the A3 alloy with a short homogenization treatment, the segregation in the matrix was obviously decreased. More Mg and Si atoms were dissolved in the matrix compared with the A2 alloy after solution treatment. Thus, the number of β″ phases and the hardness were higher than that of the A2 alloy, while the concentration of supersaturated Mg and Si atoms was lower than that in the A1 alloy due to the segregation. The hardness was slightly lower compared to the A1 alloy (Figure 9c and Figure 12b).

## 5. Conclusions

In this study, the corresponding microstructure, precipitates behavior, and mechanical properties of an age-hardened Al-Mg-Si alloy sheet fabricated by conventional DC casting, TRC, with and without homogenization heat treatment, are revealed in detail. The results will help to optimize the process of high-quality and low-cost automotive Al-Mg-Si alloy sheets. The main conclusions can be summarized as follows:

(1) The size of recrystallized grains for the DC casting alloy was marked by finely dispersed particles. With the segregated particles formed in TRC alloy, more coarse particles acted as nucleate particles to increase the recrystallization nucleation rate and dramatically decrease the recrystallized grain size.
(2) Uniformly dispersed second particles resulted in the strong Cube and R texture formed after solution treatment. The value of *r* was low due to the high volume fraction of the Cube texture for DC casting alloy. A large number of segregation particles and coarse second phase particles formed in the TRC alloy resulted in a significant PSN effect. The typical Cube_ND_ texture accompanied by the P texture made the value of *r* higher.
(3) When providing sufficient supersaturated solute atoms, more GP zones formed in the TRC alloy during T8X treatment. The strength of the TRC alloy was higher than that of the DC casting alloy. The ability of paint-bake hardening for the TRC alloy was better than DC casting alloy.
(4) The number density and volume fraction of the β″ phase were highest for the DC casting alloy. A few segregation particles that remained in the alloy decreased the concentration of supersaturated atoms, resulting in the hardness of TRC alloy being lower compared to DC casting alloy.

## Figures and Tables

**Table 1:** Intensity and volume fraction of recrystallization texture components of A1, A2, and A3 alloys after solution treatment obtained from the ODF maps.

Alloy	Component	Intensity	Volume Fraction (%)
A1	Cube	5.8	12.8
	R	1.3	5.9
A2	Cube	4.8	8.5
	Cube_ND_	2.7	12.9
	P {011} <122>	1.3	5.1
A3	Cube	4.0	9.4
	Cube_ND_	2.1	12.7
	H {001} <110>	1.3	2.1

**Table 2:** The statistics of β″ precipitates in A1, A2, and A3 alloys after paint-bake hardening at 185 °C for 8 h.

Alloy	Number Density (N/μm^2^)	Average Length/nm	Volume Fraction/%
A1	3.11 × 10^3^	22.55	2.26
A2	1.27 × 10^3^	23.71	0.74
A3	2.06 × 10^3^	18.67	1.32

**Table 3:** Calculated $\bar{r}$ values of typical recrystallized texture components [26].

Designation	Miller Indices {h k l} <u v w>	$\bar{r}$
Cube	{001} <100>	0.5
Goss	{011} <100>	15
CubeND	{001} <310>	0.5∼1
H	{001} <110>	0.5
R	{124} <211>	1.9
P	{011} <122>	2.8

## Data Availability

The processed data required to reproduce these findings cannot be shared at this time, as the data also comprise a part of an ongoing study.
